# Diagnostic role of endoscopic ultrasound-guided fine-needle aspiration (EUS-FNA) in abdominal lymphadenopathy of unknown etiology

**DOI:** 10.3389/fmed.2023.1221085

**Published:** 2023-08-31

**Authors:** Wenli Wang, Chaoqun Han, Xin Ling, Xianwen Guo, Jun Liu, Rong Lin, Zhen Ding

**Affiliations:** ^1^Division of Gastroenterology, Union Hospital, Tongji Medical College, Huazhong University of Science and Technology, Wuhan, China; ^2^Division of Gastroenterology, The First Affiliated Hospital, Sun Yat-sen University, Guangzhou, China

**Keywords:** endoscopic ultrasound-guided fine needle aspiration, lymphadenopathy, lymphoma, metastatic tumor, tuberculosis

## Abstract

**Background:**

Endoscopic ultrasound-guided fine needle aspiration (EUS-FNA) is an established method for the evaluation of abdominal organ lesions. However, there are few studies on EUS-FNA for abdominal lymph node (LN) lesions. The purpose of this study was to evaluate the diagnostic role of EUS-FNA in isolated abdominal lymphadenopathy (LAP).

**Methods:**

A retrospective analysis was performed on patients with isolated abdominal LAP who underwent a EUS-FNA examination. The diagnosis was made based on cytology, histology, and immunohistochemical (IHC) studies. The area under curve (AUC) value, sensitivity, specificity, positive predictive value (PPV), negative predictive value (NPV), and accuracy were calculated.

**Results:**

A total of 99 patients were included in this study. The final diagnoses were metastatic tumor (*n* = 32), lymphoma (*n* = 32), tuberculosis (*n* = 17), sarcoidosis (*n* = 5), castleman’s disease (*n* = 1), and reactive LAP (*n* = 12). The AUC value, sensitivity, specificity, PPV, NPV, and accuracy of EUS-FNA in the diagnosis of malignant LAP were 0.9531, 90.6, 100, 100, 85.4, and 93.9%, respectively. For the diagnosis of lymphoma, the accuracy of EUS-FNA combined with IHC staining was 94.9%. Retroperitoneal LN enlargement is more commonly associated with lymphoma, while hepatic hilar LN enlargement predominantly suggests benign conditions or metastatic tumors. Malignant lymph nodes are more likely to be regular border, circular/quasi-circular, and fusion. Lymphomas are more likely to present with fusion and heterogeneous echogenicity than metastatic tumors.

**Conclusion:**

EUS-FNA is a safe and effective method to diagnose isolated abdominal LAP.

## Introduction

In clinical practice, distinguishing between benign and malignant isolated abdominal lymphadenopathy (LAP) can be challenging, particularly when the primary lesion is not evident and imaging diagnosis is difficult (1, 2). Benign and malignant presentations of LAP include tuberculosis (TB), sarcoidosis, Castleman’s disease, Wegener’s granuloma, various rare infections, lymphoma, leukemia, and metastatic tumors, including include lung cancer, colon cancer, pancreatic cancer, and testicular cancer, among others (3–5).

Significant lesions can aid in the diagnosis, and isolated LAP presents a diagnostic problem. Conventional imaging exams, such as ultrasound, computed tomography (CT), and magnetic resonance imaging (MRI), can detect lesions, but they cannot differentiate between the types of lesions, and their diagnostic utility is limited. Positron emission tomography-computed tomography (PET-CT) is an imaging technique that combines anatomical and functional imaging (3). It can measure the cellular metabolic activity of lesions and considerably improve its ability to identify malignant lesions with the aid of tracers (such as 18F-FDG). However, tracer buildup may occur in several current infections and granulomatous illnesses, resulting in false positives that impact lesion type assessment (4, 5). Therefore, percutaneous and surgical tissue acquisition is essential to provide a pathological diagnosis.

Endoscopic ultrasound-guided fine-needle aspiration (EUS-FNA) is a diagnostic method developed in the 1990s (6, 7) that allows for real-time ultrasound (US)-guided sampling of target lesions to obtain cytological or histological criteria. In combination with immunocytochemistry (ICC), immunohistochemistry (IHC), or fluorescence *in situ* hybridization (FISH), the properties of the puncture samples can be further determined. Due to its minimally invasive and accuracy, EUS-FNA has gradually become an effective method for the diagnosis of abdominal lymph node lesions. However, in the present clinical context, there is still a paucity of research regarding the diagnostic efficacy of EUS-FNA for isolated abdominal LAP, and many medical centers continue to favor surgical biopsy for the diagnosis of lymphoproliferative diseases. In this study, we evaluated the effectiveness of the EUS-FNA diagnosis of isolated abdominal LAP.

## Materials and methods

### Patients

This retrospective study analyzed 99 inpatients who underwent EUS-FNA at the Digestive Endoscopy Center of Wuhan Union Medical College Hospital between May 2011 and December 2022 due to abdominal lymph node lesions. All patients provided written informed consent to undergo EUS-FNA, and for patients under 18 years old, informed consent was obtained from their guardians. The inclusion criteria were as follows: (1) presence of abdominal LAP (>10 mm in Diameter) indicated by at least one preoperative ultrasound, CT, MRI, or PET-CT examination, (2) no absence of parenchymal or cystic lesions with focal thickening of organ walls observed on US, CT, MRI, or PET-CT, and (3) complete diagnosis and treatment information available. Exclusion Criteria were as follows: (1) presence of organ lesions on imaging or EUS examination, (2) inability to tolerate endoscopic operation due to complications with important organs such as heart or lung, (3) serious abnormalities in coagulation function that cannot be improved after symptomatic treatment, and (4) other conditions that cannot be matched with endoscopy. The study adhered to the Helsinki Declaration, which was amended in Brazil in 2013, and approval was obtained from the Ethics Committee of Tongji Medical College, Huazhong University of Science and Technology. All patients consented to be evaluated for the study and had access to the study data.

### EUS-FNA process

#### Pre-operation

Prior to EUS-FNA procedure, preoperative evaluation of coagulation function and electrocardiogram (ECG) should be performed to exclude potential allergies to anesthetic medications. Before the procedure, patients taking oral anticoagulants should discontinue their medication for 7 days, and the patient should be told to fast before the examination.

#### In operation

The patient lay on the examination bed in the left lateral decubitus position, venous access was established in the right upper limb, and intravenous anesthesia was administered. ECG monitoring was connected to monitor the patient’s vital signs. After the endoscopist reviewed the patient’s application form, an endoscopic ultrasound (GF-UCT 2000, GF-UCT 240, and GF-UCT 260; Olympus Corporation, Tokyo, Japan) was inserted and the target lesions were examined. This included an evaluation of the lesion location, number, size, morphological characteristics, echo intensity, internal blood flow, and whether the abdominal vessels were wrapped and the surrounding organs. Elastic imaging was performed if necessary and relevant values were recorded. Once the scan was complete, the endoscopic body was adjusted and the best puncture point (Cook 19/22G, Cook Medical Inc., Bloomington, IN, United States) was selected under the supervision of ultrasonic imaging. Before puncture, Doppler exploration was performed to confirm to avoidance of peripheral blood vessels, to minimize post-puncture bleeding. The puncture needle was then connected to 5 mL/10 mL negative pressure or zero negative pressure slow pull suction. The entire puncture process was carried out under the guidance of ultrasonic images to ensure that the needle tip was quickly and accurately inserted into the lesion. After the successful puncture, the negative pressure was released, and the needle tip was withdrawn and removed. The puncture site was observed, and the procedure was concluded after confirming no bleeding. The samples obtained by puncture were slowly injected onto a clean slide, and tissue strips with a complete shape were selected for implanting into Sample bottles and fixed with 10% formaldehyde solution and sent to the Department of Pathology for further paraffin embedding section and HE staining. For those cases in which the pathological type or tissue typing could not be determined by conventional staining, IHC or FISH was performed. If the remaining sample on the slide was mostly solid, it was pressed directly and sent for cytological examination. If the blood component was more predominant, the remaining sample was saved and sent for liquid-based cytology. EUS-FNA was independently performed by two senior endoscopists in the Digestive Endoscopy Center. The procedure adhered to the highest standards of medical practice and was carried out with the utmost care to ensure patient safety and accurate diagnostic results.

#### After the operation

Following the puncture, the inpatient was sent back to the ward and given acid suppression and stomach protection medicines to prevent excess gastric acid from injuring the puncture site. The patient’s postoperative status was continuously followed, and they were urged to call their physician immediately if any symptoms such as fever, stomach discomfort, or black stool arose.

### Data collection

Participant demographics, clinical presentation, imaging report, Laboratory test results, Endoscopic ultrasound (EUS) report, pathology results, and clinical course were collected. EUS was utilized to assess the shape, size, echogenicity, boundary, and location of lymph nodes, and the diameter of the needle and the number of passes were recorded.

### Final diagnosis

The ultimate diagnosis of LAP is established based on EUS-FNA findings, long-term follow-up, and surgical pathological diagnosis (if performed). The diagnostic criteria for malignancy include the presence of malignant cytology, histology, and IHC findings in specimens obtained through EUS-FNA, percutaneous biopsy, or surgery. Additionally, evidence of metastasis in long-term follow-up, combined with clinical course and imaging, can also indicate malignancy. In cases where no malignant cells are detected through EUS-FNA, the lesion can be considered benign if there is no evidence of malignant progression in the clinical history or imaging during the follow-up period. In this study, patients were classified into malignant and benign LAP groups, all patients were followed up every 6 months, with the time of death for deceased patients serving as the endpoint of follow-up. All follow-ups were completed by June 2023.

### Statistical analysis

In this study, the results of EUS-FNA were compared with the final diagnosis to evaluate the diagnostic performance of EUS-FNA using the area under the curve (AUC), sensitivity, specificity, positive predictive value (PPV), negative predictive value (NPV), and accuracy. Categorical variables were expressed as percentages, while Qualitative variables were presented as counts and percentages, continuous variables were described as mean ± standard deviation (SD) or median (interquartile range, IQR) and compared using Student’s t-test or Mann–Whitney U test, as appropriate. According to the distribution of the data, group comparisons were conducted using the χ2 test or Fisher exact probability method. *p*-value of <0.05 was considered statistically significant. The data were collected using SPSS (version 27; IBM Corp, Armonk, New York, United States).

## Results

### Patient characteristics

**Figure 1:**
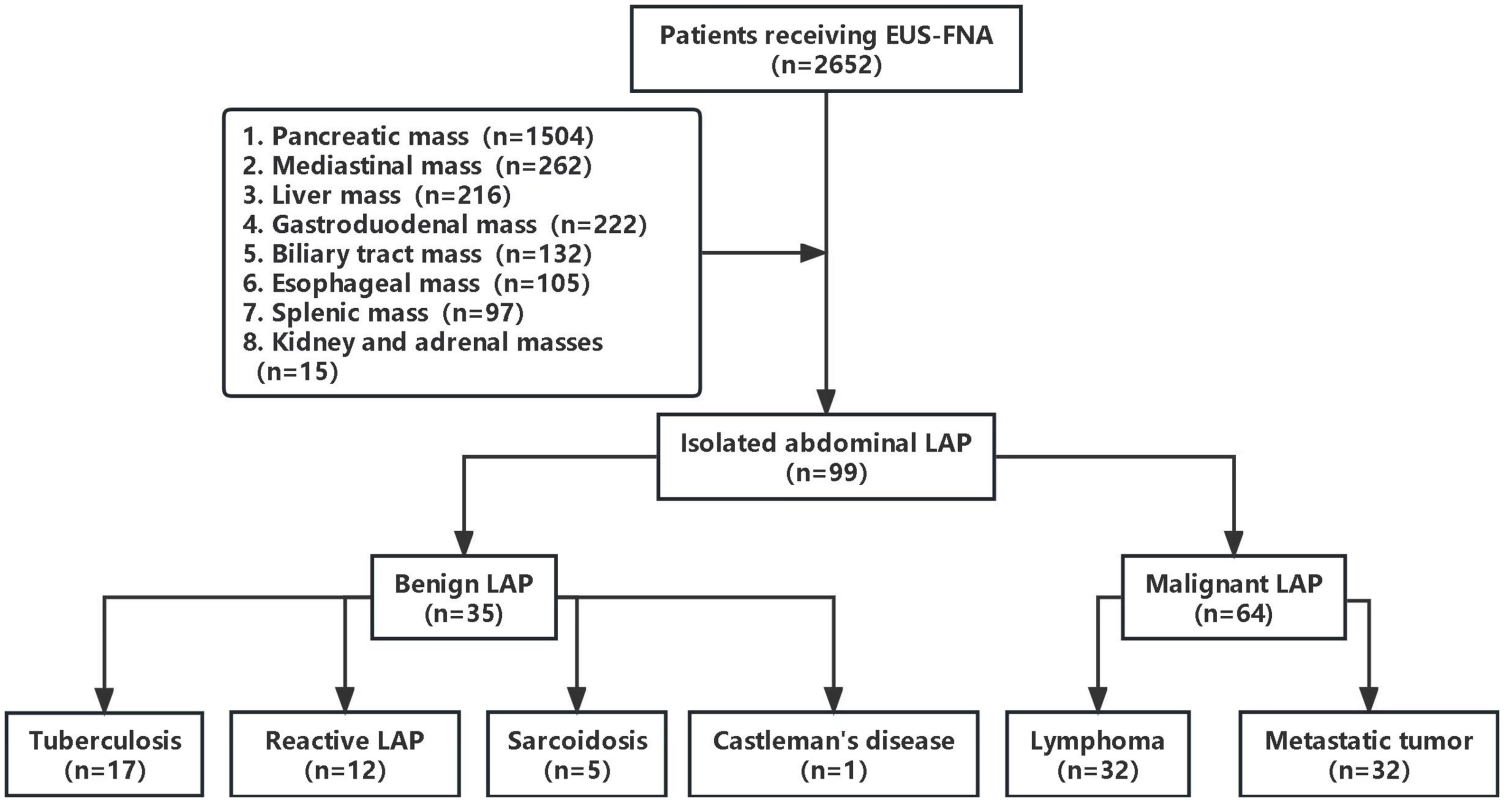
Flowchart of the patient selection process.

During the study period, 2,652 patients underwent EUS-FNA examination, retrieved from the EUS database. However, 2,553 patients were excluded due to various conditions, such as concurrent solid pancreatic masses, pancreatic cystic lesions, periampullary masses, esophageal masses, subepithelial lesions, mediastinal masses, liver and biliary masses, splenic masses, renal masses, and adrenal masses (Figure 1). Ninety-nine patients were included in this study, including 49 males and 50 females. The male-to-female ratio was 0.98:1. The age of the patients ranged from 13 to 76 years old, with an average age of 52.71 ± 12.97 years old. The majority of the enrolled patients sought therapy for gastrointestinal issues, the main clinical manifestations were abdominal pain (40.4%), some patients have no obvious symptoms or signs. Table 1 showed the baseline characteristics of the patients.

**Table 1:** General information and clinical data of patients.

Variable	Case number (*n* = 99)	Proportion (%)
*Sex*		
Male	49	49.5%
Female	50	50.5%
*Symptom*		
Abdominal pain	40	40.4%
Abdominal distension	17	17.2%
Fever	15	15.2%
No special discomfort	14	14.1%
Emaciation	12	12.1%
Fatigue	9	9.1%
Acid reflux and belching	6	6.1%
Poor appetite	6	6.1%
Chest pain	5	5.1%
Jaundice	4	4.0%
Lumbago	4	4.0%
Tawny urine	4	4.0%
Back pain	3	3.0%
Night sweats	3	3.0%
Black stool	3	3.0%
Constipation	2	2.0%
Hiccup	2	2.0%
Limb numbness	2	2.0%
Cough	2	2.0%
Gasp	1	1.0%
Diarrhea	1	1.0%
Dizziness	1	1.0%
Skin pruritus	1	1.0%
Joint pain	1	1.0%
*Sign*		
No obvious signs	35	35.4%
Abdominal tenderness	15	15.2%
Skin sclera yellow staining	5	5.1%
Superficial lymph node enlargement	5	5.1%
Ascites sign	3	3.0%
Elevated abdominal muscle tone	2	2.0%
Anemic appearance	2	2.0%
Splenomegaly	2	2.0%
Pleural effusion	1	1.0%
Abdominal mass	1	1.0%
Tenderness of the liver region	1	1.0%
Lower limb edema	1	1.0%
*Aspiration needle size, n (%)*		
19-gauge	65	65.7%
22-gauge	34	34.3%
*Mean number of puncture*	2.5 ± 1.0	
*Lesion type*		
Malignant	64	64.6%
Benign	35	35.4%

### Final diagnosis

Based on surgical pathology (*n* = 19) and long-term follow-up, the final diagnoses included 35 cases of benign LAP and 64 cases of malignant LAP. Among them, there were 32 cases of lymphoma and 32 cases of metastatic tumors, 17 cases of TB, 12 cases of reactive LAP, 5 case of sarcoidosis and 1 case of Castleman’s disease (Table 2).

**Table 2:** Final diagnosis of undiagnosed LAP.

Diagnosis	Number of cases (*n* = 99)	Proportion (%)
Lymphoma	32	32.3%
Diffuse large B-cell lymphoma	16	16.2%
Follicular lymphoma	6	6.1%
Angioimmunoblastic T cell Lymphoma	4	4.0%
Classical Hodgkin lymphoma	2	2.0%
NK/T cell lymphoma	1	1.0%
Unclassified	3	3.0%
Metastatic cancer	32	32.3%
Colon cancer	5	5.1%
Gastric cancer	5	5.1%
GallbLAPder cancer	4	4.0%
Liver cancer	4	4.0%
The primary tumor was not identified	3	3.0%
Pancreatic cancer	3	3.0%
Cholangiocarcinoma	2	2.0%
Thymic carcinoma	2	2.0%
Rectal cancer	1	1.0%
Hepatoid adenocarcinoma	1	1.0%
Gastrointestinal stromal tumor	1	1.0%
Lung cancer	1	1.0%
Tuberculosis	17	17.2%
Sarcoidosis	5	5.1%
Castleman’s disease	1	1.0%
Reactive lymphadenopathy	12	12.1%

The main cause of LAP is malignant tumor (64.6%). In this study, eight patients had a history of tumors, and EUS-FNA confirmed the recurrence of malignant tumors. The average time to recurrence after primary tumor resection was 3.1 years (ranging from 7 months to 8 years). EUS-FNA diagnosed the primary lesions of metastatic tumors in 23 cases, and subsequently confirmed by PET-CT or surgical biopsy. Among them, the most common colon cancer (*n* = 5), gastric cancer (*n* = 5), gallbLAPder cancer (*n* = 4), liver cancer (*n* = 4). Twenty-six cases were diagnosed with lymphoma using EUS-FNA, 6 undiagnosed patients were confirmed by US-guided and CT-guided percutaneous puncture after a follow-up visit. Finally, 29 lymphomas were correctly classified according to the 2016 World Health Organization Classification Criteria for lymphoid tumors (6), including 16 cases of diffuse large B-cell lymphoma (DLBCL), 6 cases of follicular lymphoma (FL), 4 cases of angioimmunoblastic T-cell lymphoma (AITL), 2 cases of Classical Hodgkin lymphoma (cHL), and 1 case of NK/T cell lymphoma (NKTL).

### Characteristics of lesions in EUS

**Figure 2:**
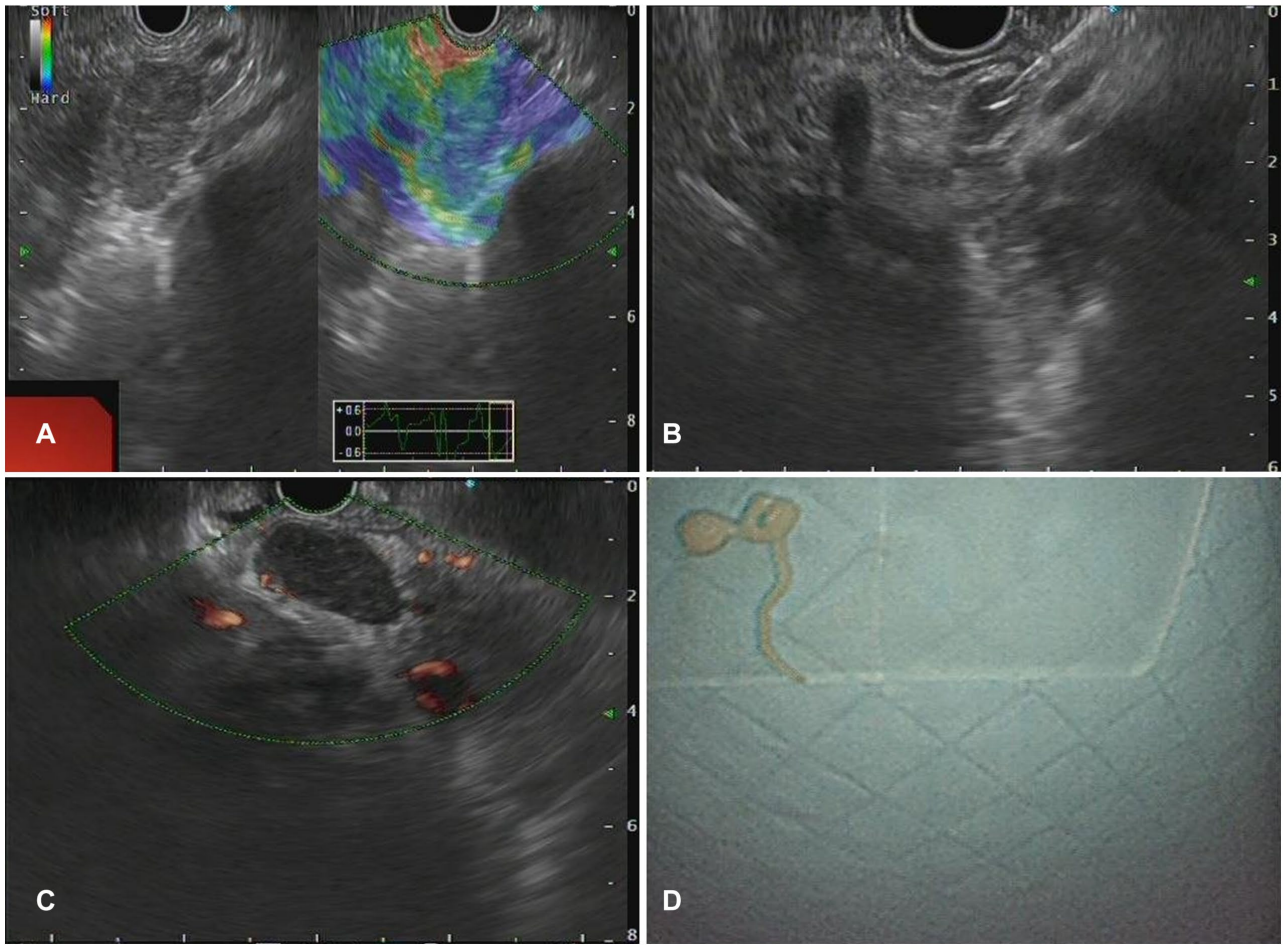
The Endoscopic ultrasonography image features of Benign Lymphadenopa-thy in patients. **(A)** A case of reactive lymphadenomegaly. Multiple hypoechoic nodules, some of which were fused into clusters, with a hard texture on elastography were observed during EUS. **(B)** A case of sarcoidosis. A low echo lymph node shadow was identified in the hepatic portal area on EUS, with no obvious blood flow signal in the lesion on Doppler, and elastography indicated a hard texture of the lesion. **(C,D)** One case of lymph node TB. EUS showed clear boundary lymph nodes with regular morphology **(C)** and pus in the specimen obtained from fine needle aspiration of a lymph node **(D)**.

**Figure 3:**
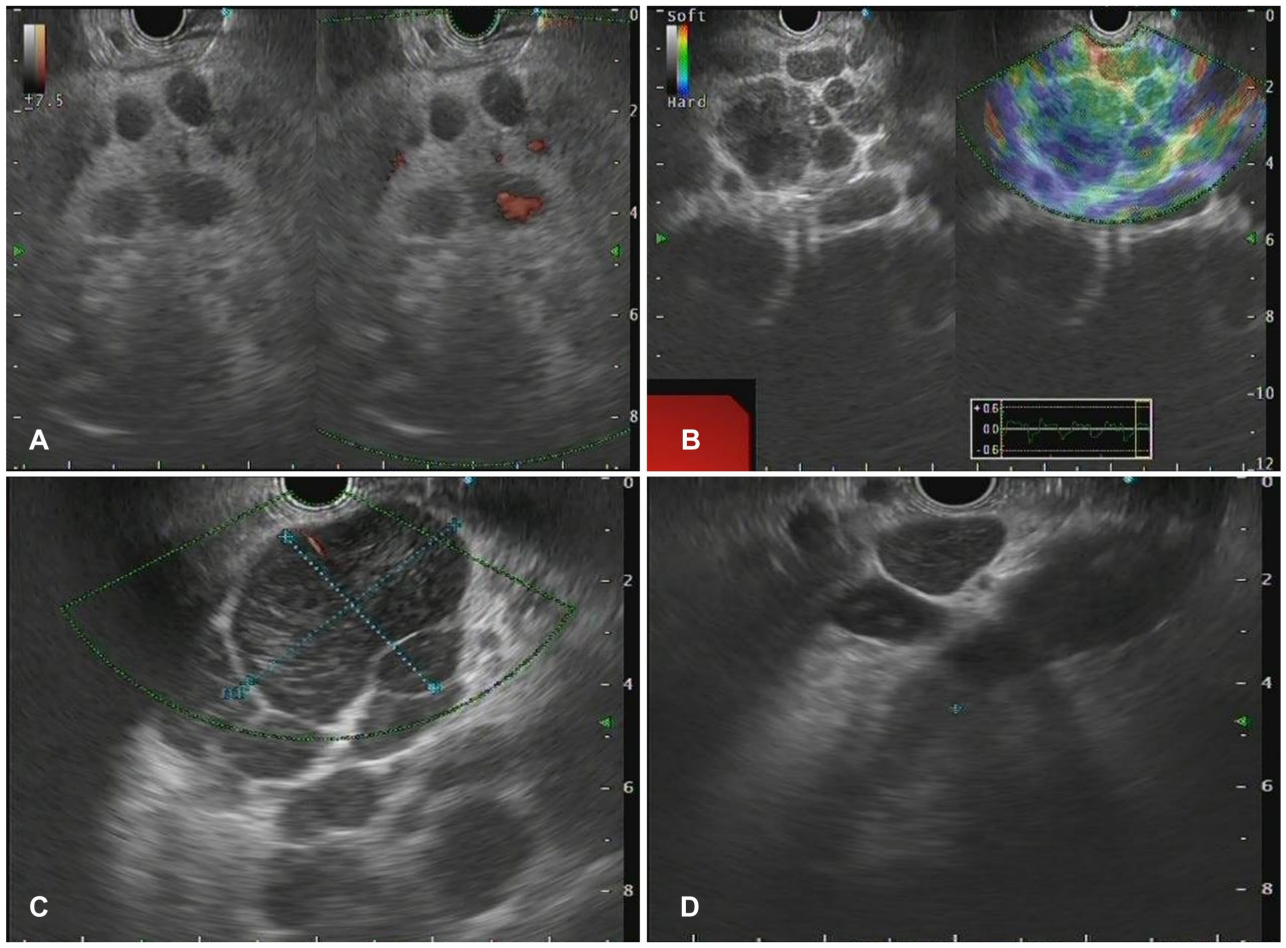
The Endoscopic ultrasonography image features for lymphoma. **(A)** A case of NKT cell lymphoma. EUS showed circular hypoechoic nodules with partial fusion, and no obvious blood flow signal was observed by Doppler. **(B)** In the case of follicular lymphoma, EUS showed multiple hypoechoic nodules and partial fusion. **(C)** One case of diffuse large B-cell lymphoma. EUS showed multiple hypoechoic nodules, Doppler showed a few blood flow signals, and elastic imaging suggested a hard texture. **(D)** A case of vascular immunoblastic T-cell lymphoma. EUS showed multiple circular hypoechoic nodules with partial fusion.

**Figure 4:**
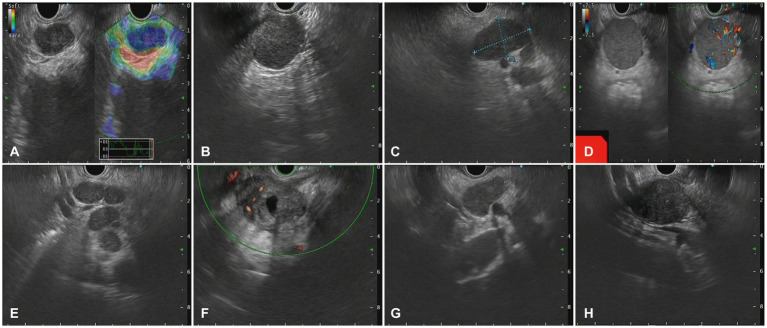
The Endoscopic ultrasonography image features in patients with metastatic tumors. **(A)** A case of cholangiocarcinoma. EUS showed multiple hypoechoic mass shadows, Doppler showed no blood flow signal, and the texture of elastic imaging was hard. **(B)** A case of gallbladder carcinoma. EUS showed low echo fusion lymph node shadow **(C)** A case of lung cancer. EUS showed multiple uniform hypoechoic nodules, some of which were fused with unclear boundaries, Doppler imaging showed no obvious blood flow signal, and the texture of elastic imaging was hard. **(D)** A case of liver cancer. EUS showed a class of circular lesions with uniform hypoechoic changes and Doppler blood flow signals. **(E)** A case of colon cancer. EUS showed multiple hypoechoic nodules, some of which were fused into clusters, with clear boundaries and homogeneous internal echoes. **(F)** A case of gastric cancer. EUS showed multiple hypoechoic nodules, which were round, with clear boundaries, anechoic, and non-uniform echoic necrosis. **(G)** A case of thymic carcinoma. EUS showed multiple enlarged lymph nodes with clear boundaries, and no obvious blood flow signal was observed by Doppler. **(H)** A case of rectal cancer. EUS scan showed circular hypoechoic lesions with partial fusion, and the Doppler scan showed no obvious blood flow signal.

The morphology of lymph node may present as circular, quasi-circular, or irregular, while the border may present as either regular or irregular. In this study, the distribution of LN morphology revealed that 61 cases (61.6%) demonstrated circular and oval shapes, whereas 38 cases (38.4%) displayed irregular shapes. Fifty-six lesions (56.6%) exhibited regular borders, while 33 lesions (33.3%) demonstrated irregular borders. Sonographic evaluation of 93 LNs (93.9%) indicated low echogenicity. EUS images of characteristic benign lesions, lymphomas, and metastatic tumors were shown in Figures 2–4.

As shown in Table 3, there was no significant difference in the incidence of malignant LAP between males and females in this study cohort (*p* = 0.6753). Malignant LAP was more prone to develop retroperitoneal LN metastasis (*p* = 0.0035), while benign LAP was more likely to involve the hepatic hilar LN (*p* = 0.0275). Compared to benign LAP, the more common characteristics of malignant LAP were Well-defined margin, Circular/quasi-circular shape, and fusion (*p* < 0.0001, *p* < 0.0001, *p* = 0.0419). There were no significant differences between the two groups in terms of lesion size, echogenicity, number of lesions, and hypoechogenicity, indicating that these echogenic features cannot serve as absolute indicators for predicting the malignancy of enlarged LNs.

**Table 3:** Patient and lesion characteristics of malignant and benign LAP.

Patient characteristics	Malignant LAP (*n* = 64)	Benign LAP (*n* = 35)	*p*-value
Sex, *n*			0.6753
Male	33	16	
Female	31	19	
Age, years^b^	54 (48, 65)	51 (42, 57)	0.0671
Lesion location^c^			
Hepatic hilar	17 (26.6)	17 (48.6)	0.0275^a^
Retroperitoneal	32 (50.0)	7 (20.0)	0.0035^a^
Paraperitoneal trunk	12 (18.7)	7 (20.0)	0.8800
Peripancreatic	3 (4.7)	4 (11.4)	0.2397
Endoscopic ultrasound characteristics			
Size, mm^b^	28.50 (8.50, 38.00)	26.00 (20.00, 31.00)	0.5847
Multifoci diseases^c^	53 (82.8)	27 (77.1)	0.4935
Well-defined margins^c^	48 (75)	8 (22.9)	<0.0001^a^
Hypoechogenic^c^	62 (96.9)	31 (88.6)	0.1811
Fusion^c^	41 (64.1)	15 (26.8)	0.0419a
Circular/quasi-circular lesion^c^	52 (81.3)	9 (25.7)	<0.0001^a^
Homogeneous echogenicity^c^	31 (48.4)	19 (54.3)	0.5779

a Statistically significant, *p* < 0.05.

b Data are presented as Median and quartile spacing.

c Data are presented as *n* (%).

As presented in Table 4, the results indicated that when malignancy was suspected, enlarged LNs in the hepatic hilum region were predominantly attributed to metastatic tumors (*p* = 0.0476). Moreover, the occurrence of lymphoma involving the retroperitoneal area was significantly higher than that of metastatic tumors (*p* = 0.0124). Furthermore, lymphoma demonstrated a higher tendency for LN fusion (*p* = 0.0007) and heterogeneous echogenicity (*p* < 0.0001). These findings provided significant insights for distinguishing between lymphoma and metastatic tumors.

**Table 4:** Patient and lesion characteristics of metastatic tumors and lymphoma.

Patient characteristics	Metastatic tumors (*n* = 32)	Lymphoma (*n* = 32)	*p*-value
Sex, *n*			0.4530
Male	18	15	
Female	14	17	
Age, years^b^	55 (48, 63.5)	54 (42, 68.75)	0.9625
Lesion location^c^			
Hepatic hilar	12 (37.5)	5 (15.6)	0.0476^a^
Retroperitoneal	11 (34.4)	21 (65.6)	0.0124^a^
Paraperitoneal trunk	7 (21.9)	5 (15.6)	0.5218
Peripancreatic	2 (6.2)	1 (3.1)	1.0000
Endoscopic ultrasound characteristics			
Size, mm^b^	27 (18.5, 33.5)	30 (18.5, 40)	0.8824
Multifoci diseases^c^	25 (78.1)	28 (87.5)	0.3202
Well defined margins^c^	24 (75.0)	24 (75.0)	1.0000
Hypoechogenic^c^	31 (96.9)	31 (96.9)	1.0000
Fusion^c^	14 (43.8)	27 (84.4)	0.0007^a^
Circula /quasi-circularr lesion^c^	26 (81.3)	26 (81.3)	1.0000
Homogeneous echogenicity^c^	24 (75.0)	7 (21.9)	<0.0001^a^

a Statistically significant, *p* < 0.05.

b Data are presented as Median and quartile spacing.

c Data are presented as *n* (%).

### Diagnostic efficacy of EUS-FNA

In this study, we present the diagnostic performance of EUS-FNA in identifying malignant LAP. The AUC value, sensitivity, and specificity were reported as 0.9531, 90.6, and 100%, respectively. Moreover, the NPV and PPV were found to be 100 and 85.4%, respectively, resulting in an overall accuracy of 93.9% (Table 5). Subsequently, the inclusion of IHC in the diagnostic process led to improved accuracy (95.1%) and an increased AUC value (0.9650). Notably, when assessing specific disease types, EUS-FNA demonstrated diagnostic accuracies of 100, 94.0, and 99.0% for metastatic tumors, lymphoma, and TB (Table 6).

**Table 5:** Diagnostic performance of different pathological methods for LAP.

Method	*N*	Final diagnoses, *n*		Sensitivity	Specificity	PPV	NPV	Accuracy	AUC
		Malignant	Benign						
Histology	99								
Malignant		58	0	90.6	100	100	85.4	93.9	0.9531
Benign		6	35						
Cytology	65								
Malignant		19	2	44.2	90.9	90.4	45.5	60.0	0.6755
Benign		24	20						
IHC staining	79								
Malignant		53	0	93.9	100	100	80	95.1	0.9650
Benign		4	22						

PPV, positive predictive value; NPV, negative predictive value.

Furthermore, our study assessed the accuracy of cytology and IHC in diagnosing lymphoma, yielding a sensitivity and accuracy of 37.5 and 58.5%, respectively. With the addition of IHC the sensitivity and accuracy increased to 86.7 and 94.9%, respectively (Supplementary Table S1). Importantly, when discrepancies between cytological and histological diagnoses were observed, histology was considered more reliable, as demonstrated in Supplementary Table S2.

The accurate diagnosis of EUS-FNA can help in the timely development of appropriate treatment plans for the disease. In this study, patients with lymphoma or metastatic tumors were to do cancer staging and treated with chemotherapy or surgery as appropriate, in cases where no primary lesion was found on imaging, further evaluation with a PET-CT scan was recommended. Patients with negative findings on EUS-FNA but presenting with clinical suspicion of TB should undergo further diagnostic investigations, including acid-fast bacilli (AFB) staining, culture of *Mycobacterium tuberculosis*, TB polymerase chain reaction (TB-PCR), and GeneXpert. These tests are particularly relevant for specific patient subgroups, such as immunocompromised individuals or certain age groups, who are at a higher risk of TB infection.

**Table 6:** Diagnostic performance of endoscopic ultrasound-guided fine-needle aspiration in the evaluation of LAP.

Definite diagnosis	Sensitivity	Specificity	PPV	NPV	Accuracy
Metastatic tumors	100	100	100	100	100
Lymphoma	81.3	100	100	91.8	94.0
Tuberculosis	94.1	100	100	98.8	99.0
Nonspecific reactive lymphadenopathy	91.7	97.7	84.6	98.8	97.0

NPV, negative predictive value; PPV, positive predictive value.

A 52-year-old female patient presented with fever, weight loss, and multiple enlarged abdominal LNs. TB-PCR and Xpert tests yielded negative results. PET-CT examination suggested a possible diagnosis of lymphoma. Subsequent EUS-FNA confirmed TB upon pathological examination. Consequently, the patient’s treatment plan was modified, and she was referred to an anti-TB center for management, resulting in complete recovery.

In 2018, one patient diagnosed with poorly differentiated gallbladder adenocarcinoma underwent postoperative chemotherapy with gemcitabine and Tiggio. In April 2019, radical gallbladder carcinoma resection and laparoscopic abdominal exploration were performed in our hospital. The patient received two cycles of Capecitabine in combination with Irinotecan (XELIRI) chemotherapy. Abnormal enhanced nodules in the primary gallbladder fossa were reviewed to assess tumor recurrence/metastasis possibility. Different chemotherapy regimens were subsequently employed to monitor disease progression in various areas, including the gallbladder fossa, portal area, anterior left lobe of the liver, omentum, and retroperitoneum.

No serious complications were reported following EUS-FNA, although one patient experienced postoperative fever, while another experienced mild abdominal pain, both of which resolved with anti-inflammatory therapy.

## Discussion

In this study, we retrospectively analyzed the diagnostic impact of EUS-FNA on abdominal isolated LAP. Although EUS-FNA is recognized as the preferred method for pancreatic lesions (8), its diagnostic value for abdominal isolated LAP has not been clearly defined.

The etiology of abdominal LAP varies according to patient characteristics and geographic location (9, 10). India primarily exhibits pulmonary TB, while lymphoma is predominant in Japan (11). Our study found that lymphoma (32.3%), metastatic tumors (32.3%), and TB (17.2%) were the main causes, which is similar to Western countries (11). Malignant LAP diagnostic criteria proposed by Catalano et al. based on EUS imaging include the presence of round or oval cross-sections, well-defined borders, hypoechoic interiors, and diameters larger than 1 cm (12). We observed similar results on isolated abdominal LAP, where malignant lesions were more likely to exhibit regular LN borders, circular or nearly circular shapes, and fusion features. Furthermore, our results showed that LAP at the hepatic hilum mostly indicated benign lesions or metastatic tumors, while retroperitoneal LNs with fusion were more associated with lymphoma, consistent with the findings of Pausawasdi et al. (1). Wang’s study (13) suggested that malignant LNs detected along the abdominal axis were more likely to be lymphoma; however, they only reported eight cases of para-aortic lymphoma, resulting in limited evidence. Contrary to the studies conducted by Laith et al. (14, 15), our study did not find a significant correlation between LN size, patient age, and malignant lesions.

Previous studies have demonstrated the utility of EUS-FNA. Nakahara et al. reported an accuracy rate of 96% in diagnosing abdominal LAP in a study involving 57 cases (4). For mediastinal LAP, EUS-FNA shows a sensitivity range of 71–94% and an accuracy range of 86–93% (16–19). However, previous studies often included cases with solid organ masses, mixed multi-site LN diseases, and had limitation in sample sizes. Therefore, to address these limitations, our study specifically focused on enlarged abdominal and retroperitoneal LNs. The results revealed that EUS-FNA exhibits high diagnostic value for isolated abdominal LAP with a sensitivity of 90.6%, specificity of 100%, accuracy of 93.9%, and an AUC value of 0.9531. These findings are consistent with prior research outcomes (18, 19).

The accurate diagnosis provided by EUS-FNA is crucial for disease management. Although PET-CT is considered a valuable tool for prediction and prognosis, its sensitivity and negative predictive value are limited (5). Therefore, pathological results remain essential in guiding clinical decision-making. In malignant tumor patients, the combination of EUS-FNA and IHC offers effective discrimination of subtypes (20), aiding in cancer staging and enabling timely administration of further treatments such as surgery, chemotherapy, or radiotherapy. For benign diseases, treatment options typically involve standard anti-TB, immunosuppressive, or anti-inflammatory therapies based on the specific lesion type. In this study, EUS-FNA demonstrated excellent diagnostic performance for specific diseases, with accuracies of 100% for metastatic tumors, 94% for lymphomas, 99% for tuberculosis, and 97% for reactive lymphoid hyperplasia.

The handling and testing methods of specimens have a significant impact on the diagnostic results of EUS-FNA. The technical guidelines by the European Society of Gastrointestinal Endoscopy (ESGE) state that smear cytology is more accurate than liquid-based cytology in diagnosing pancreatic masses and suspicious lymph node aspirates (20). However, Hashimoto et al. (21) have presented conflicting conclusions. In this study, among the 65 cases that underwent smear cytology, the sensitivity and accuracy for diagnosing benign and malignant lesions were 44.2 and 60%, respectively, with an AUC value of 0.6755, significantly lower than histolog (22). The reasons for this may include inadequate sample volume obtained through aspiration, susceptibility to blood contamination, cell stacking, and cell damage caused by manual slide preparation (23). Combining various pathological methods can improve diagnostic accuracy. For example, in diagnosing lymphoma, when there are abundant reactive lymphoid cells or a mixture of tumor cells, we can improve sample quality through rapid on-site evaluation (ROSE) and combine IHC staining and flow cytometry (FCM) to reduce the probability of misdiagnosis and missed diagnosis. Ribeiro et al. found that combining EUS-FNA cytology with flow cytometry (FC) or ICC increased diagnostic accuracy by 31% (24). In this study, the combination of EUS-FNA and IHC improved the diagnostic accuracy of lymphoma from 58.5 to 93.3%. Currently, there is limited research on the impact of ROSE on the diagnosis of LAP by EUS-FNA, and existing studies have contradictory conclusions (25–27). Ganc et al. (28) demonstrated that ROSE increased the diagnostic accuracy of EUS-FNA from 79 to 96%. In a recent multicenter randomized controlled trial (29), the implementation of ROSE during EUS-FNA did not increase the diagnostic yield for mediastinal and abdominal lymph node lesions. The decision to perform ROSE should be based on a comprehensive consideration of cost and output factors by the hospital administration.

In recent years, endoscopic ultrasound-guided fine-needle biopsy (EUS-FNB) devices utilizing core biopsy have been developed and are considered to provide superior tissue sampling compared to FNA (30–32). A recent meta-analysis comparing EUS-FNB with standard EUS-FNA demonstrated no significant differences between the two needles in terms of sample adequacy, diagnostic accuracy, or core specimen collection (33). Nevertheless, FNB is associated with higher risks of infection and bleeding (34), thus our hospital only utilizes FNA for the diagnosis of abdominal LN lesions. EUS-FNA has a certain rate of false-negative results, particularly for T-cell lymphomas where IHC has limited diagnostic utility. Thus, cases of this nature require open biopsy for further confirmation and disease classification purposes (24). In our study, two patients exhibited false-negative results in IHC, which were subsequently confirmed as lymphomas through surgical biopsy. Previously, it was believed that the quantity of tissue obtained *via* aspiration significantly impacted the positivity rate of the procedure. Some studies indicate that smaller gauge needles (22G or 25G) provide smaller material volumes compared to larger gauge needles (19G), yet they are associated with reduced bleeding risk and increased maneuverability, thereby allowing for more accurate diagnoses (35–37). The 2017 ESGE sampling guidelines recommend utilizing a 25G or 22G needle for LN sampling (20). In our research, we did not observe any differences in diagnostic accuracy between the use of 19G or 22G needles (*p* = 0.1771). Additionally, we did not identify any bleeding incidents associated with the use of larger gauge needles in the medical records.

One of the primary limitations of this study is the scarcity of cases related to isolated abdominal LAP, which makes it challenging to obtain a large sample size for retrospective data analysis. Additionally, the earliest cases collected in this study date back to 2011, and the diagnostic capability of EUS-FNA continues to improve. Retrospective studies are unable to accurately control for variations in operator skill level and imaging equipment consistency. Therefore, further multicenter prospective studies are warranted to ascertain the diagnostic value of EUS-FNA for isolated abdominal LAP.

In summary, EUS-FNA is a reliable diagnostic method for lymphoma, metastatic carcinoma, and tuberculosis, effectively avoiding unnecessary surgeries.

## Data availability statement

The original contributions presented in the study are included in the article/Supplementary material, further inquiries can be directed to the corresponding authors.

## Ethics statement

The studies involving humans were approved by Medical Ethics Committee of Tongji Medical College, Huazhong University of Science and Technology. The studies were conducted in accordance with the local legislation and institutional requirements. The participants provided their written informed consent to participate in this study.

## Author contributions

WW and CH designed the study, collected medical records data, analyzed the data, and drafted the manuscript. XL, XG, and JL contributed to data collection. ZD and RL supervised the study and revised the manuscript. All authors contributed to the article and approved the submitted version.

## Funding

This study was supported in part by the National Natural Science Foundation of China (No. 82070667).

## Conflict of interest

The authors declare that the research was conducted in the absence of any commercial or financial relationships that could be construed as a potential conflict of interest.

## Publisher’s note

All claims expressed in this article are solely those of the authors and do not necessarily represent those of their affiliated organizations, or those of the publisher, the editors and the reviewers. Any product that may be evaluated in this article, or claim that may be made by its manufacturer, is not guaranteed or endorsed by the publisher.
