# Supplementary material for: Diagnostic role of endoscopic ultrasound-guided fine-needle aspiration (EUS-FNA) in abdominal lymphadenopathy of unknown etiology
Source: Front Med (Lausanne). 2023 Aug 31;10:1221085. doi: 10.3389/fmed.2023.1221085 (PMC10501754; doi:10.3389/fmed.2023.1221085)
Supplement: Supplementary file 1 [file Data_Sheet_1.docx]

Supplementary Material

Diagnostic Role of Endoscopic Ultrasound-Guided Fine-Needle Aspiration in Abdominal Lymphadenopathy of Unknown Etiology

Wenli Wang^1†^, Chaoqun Han^1†^, Xin Ling^1^, Xianwen Guo^1^, Jun Liu^1^, Rong Lin^1*^, Zhen Ding^1*,2^

*** Correspondence:** Rong Lin: [selinalin35@hotmail.com](mailto:selinalin35@hotmail.com); Zhen Ding: dingzh26@mail.sysu.edu.cn.

# Supplementary Tables

**Supplementary table 1**. Diagnostic efficacy of different pathologic diagnostic methods for lymphoma

| Method | N | [golden standard](javascript:;) | | Sensitivity | Specificity | PPV | NPV | Accuracy |
| --- | --- | --- | --- | --- | --- | --- | --- | --- |
|  |  | lymphadenopathy | Non-lymphoma |  |  |  |  |  |
| Cytology | 65 |  |  |  |  |  |  |  |
| lymphadenopathy |  | 9 | 12 | 37.5 | 70.7 | 42.9 | 65.9 | 58.5 |
| Non-lymphoma |  | 15 | 29 |  |  |  |  |  |
| IHC | 79 |  |  |  |  |  |  |  |
| lymphadenopathy |  | 26 | 0 | 86.7 | 100 | 100 | 92.5 | 94.9 |
| Non-lymphoma |  | 4 | 49 |  |  |  |  |  |
| PPV, positive predictive value; NPV, negative predictive value | | | | | | | | |

**Supplementary table 2**. Inconsistent cytological with histological diagnosis

| Sex | Age | Cytological  diagnosis | Histological diagnosis | IHC results |
| --- | --- | --- | --- | --- |
| Female | 74 | False negative | DLBCL | CD20+, CD3-, CD10-, BCL6-, CD5-, CD56-, P53-, CD30-, Ki67 (Li:90%) |
| Female | 70 | False negative | DLBCL | CD20+, CD3-, PAX5+, CD19+, CD22+, CD10-, BCL6+, MUM1+, BCL2+, C-Myc (40%+), CD5-, CD21+, CD30-, CycLinD1-, P53 (20%+), Ki67 (Li:80%) |
| Male | 72 | False negative | DLBCL | CD45+, CD20+, CD3-, CD34-, Ki67 (Li:90%) |
| Male | 49 | False negative | DLBCL | CD20+, CD3-, CD5-, CD10, CD19+, BCL6+, Ki67 (Li:80%) |
| Male | 38 | False negative | FL | CD3-, CD20+, CD10+, Ki67 (Li:50%) |
| Female | 54 | False negative | AITL | CD3+, CD2+, CD7+, CD5+, CD43+, CD21+, BCL6+, BCL2+, Ki67 (Li:40%) |
| Male | 67 | False negative | Thymoma | PCK+, CK5/6+, P63+, P40+, CD5+, CD117+, S100-, CD20-, TdT-, Ki67 (Li:60%) |
| Male | 65 | False negative | Neuroendocrine carcinoma | PCK+, Syn+, CD56+ |
| Male | 51 | False negative | [Lung cancer](javascript:;) | PCK+, CK7+, CK20+, VilLin+, CDX2+ |
| Male | 55 | False negative | Liver cancer | PCK+, CK8/18+, CK7+, CK20-, VilLin-, Calretinin±, Syn-, CgA-, S100-, Glypican-3-, Hepa-, AFP-, TTF-1-, PAX8-, GATA3-, CD34-, WT-1-, ERG-, Ki67 (Li:50%) |
| Male | 68 | False negative | Cancer of unknown primary | PCK+, CK8/18+, CK7+, C-Myc (Li:80%) |
| Male | 40 | False negative | FL | CD20+, CD3-, CD10+, BCL6+, MUM1-, BCL2+, HGAL+, CD21+, CD30-, CD5-, CycLinD1-, C-Myc (5%+), P53 (30%+), Ki67 (Li:10%), EBER- |
| Female | 71 | False negative | [Gastrointestinal stromal tumor](javascript:;) | CD117+, CD34+, DOG-1+, SMA-, S100-, Ki67 (Li:5%) |
| Male | 64 | False negative | [Gastric carcinoma](javascript:;) | Tubularadeno carcinoma HER2 2+, Mucinousadeno carcinoma HER2 1+, Signetringcell carcinoma HER2 0 |
| Female | 73 | False negative | DLBCL | CD20+, CD19+, CD22+, CD38+, CD3-, CD21-, CD30-, CD10+, BCL6+, MUM1-, CD5-, CycLinD1-, BCL2-, C-Myc (60%+), P53 (40%+), TdT-, PD1-, Ki67 (Li:90%) |
| Male | 42 | False negative | FL | CD20+, CD21+, CD10+, BCL6+, MUM1-, BCL2+, CD23+, CD5-, CycLinD1-, CD43-, Kappa-, Lambda-, IgM+, IgD+, CD30-, HGAL+, CD3-, PCK-, Ki67 (Li:10%) |
| Female | 29 | False negative | FL | CD20+, PAX5+, CD3+, CD5+, CD10+, BCL6+, CD21+, CD23+, CD200+, BCL2+, CD30-, TdT-, MUM1-, CycLinD1-, EBV:EBER- |
| Female | 60 | False negative | Neuroendocrine carcinoma | PCK+, CK8/18+, PGP9.5+, CD56+, CK7+, VilLin+, CgA-, Syn-, P63-, CK5/6-, LCA-, S100-, Desmin-, WT-1-, CK20-, TTF-1-, CDX2-, GATA-3- |
| Male | 61 | False negative | [Gastric carcinoma](javascript:;) | PCK+, P40+, CK7+, CK20-, VilLin-, CDX2+, TTF-1-, PSA-, PAX2-, GATA-3-, Hepatocyte-, Glypican-3- |
| Male | 60 | False negative | Angioimmunoblastic T cell Lymphoma | CD3+, CD4+, CD8-, CXCL13+, PD1+, CD2+, CD5+, CD7+, TIA1+, GranB+, CD56-, MUM1+, CD21+, CD20-, CD10-, BCL6-, HGAL-, Ki67 (Li:50%) |
| Female | 54 | False negative | DLBCL | CD20+, CD3-, BCL6+, MUM1+, PAX5+, BcL2+, CD21-, CD30-, ALK-, CD10-, PCK-, C-myc (60%), Ki67 (Li:80%) |
